# Medicaid Spending in Coordination-Only Dual-Eligible Special Needs Plans

**DOI:** 10.1001/jamanetworkopen.2024.55461

**Published:** 2025-01-22

**Authors:** Neto Coulibaly, Kelley A. Jones, Valerie A. Smith, Megan Shepherd-Banigan, Michelle Scotton Franklin, Courtney H. Van Houtven, M. Kate Bundorf, Brystana G. Kaufman

**Affiliations:** 1 Brown University School of Public Health, Brown University, Providence, Rhode Island; 2 Department of Population Health Sciences, Duke University School of Medicine, Durham, North Carolina; 3 Center of Innovation to Accelerate Discovery and Practice Transformation, Durham VA Medical Center, Durham, North Carolina; 4 Division of General Internal Medicine, Department of Medicine, Duke University, Durham, North Carolina; 5 Department of Biostatistics and Bioinformatics, Duke University, Durham, North Carolina; 6 Duke Margolis Institute for Health Policy, Duke University, Durham, North Carolina; 7 VA Mid-Atlantic Mental Illness Research Education and Clinical Care, Durham VA Health Care System, Durham, North Carolina; 8 Department of Psychiatry and Behavioral Sciences, School of Medicine, Duke University, Durham, North Carolina; 9 Sanford School for Public Health Policy, Duke University, Durham, North Carolina

## Abstract

**Importance:**

More than 4 million Medicare beneficiaries have enrolled in dual-eligible Special Needs Plans (D-SNPs), and coordination-only D-SNPs are common. Little is known about the impact of coordination-only D-SNPs on Medicaid-covered services and spending, including long-term services and supports, which are financed primarily by Medicaid.

**Objective:**

To evaluate changes in Medicaid fee-for-service (FFS) spending before and after new enrollment in coordination-only D-SNPs vs new enrollment in non–D-SNP Medicare Advantage (MA) plans among community-living beneficiaries enrolled in both Medicare and North Carolina Medicaid.

**Design, Setting, and Participants:**

This cohort study applied a new user, active comparator design to control for selection into MA and inverse probability of treatment weighting to improve the comparability between groups. The cohort included community-living dual-eligible Medicare and Medicaid beneficiaries in North Carolina with 365 days of Medicare FFS enrollment prior to new enrollment in D-SNP (treatment) or other MA plan (active comparator). Linked 100% Medicare and North Carolina Medicaid claims data (2014-2017) provided payments across both payers prior to MA enrollment; after MA enrollment, payments for Medicaid-funded services and supplemental Medicaid payments for Medicare-funded services were observed. Data were analyzed from August 2023 to November 2024.

**Exposure:**

New D-SNP enrollment.

**Main Outcomes and Measures:**

Outcomes included annualized 1-year Medicaid FFS spending overall and by claim type, including inpatient, outpatient, carrier, home health, personal care services, and behavioral health services.

**Results:**

Among 8869 participants in the D-SNP cohort, 4762 (53.7%) were younger than 65 years, 5833 (65.8%), were female, and 975 (11.0%) resided in rural areas. After inverse probability of treatment weighting, characteristics were similar among the comparison MA cohort of 4389 participants (4706 [53.2%] aged <65 years; 5739 [64.9%] female; 971 [11.0%] rural). There were no significant differences in Medicaid FFS spending per person-year (PPY) at baseline or differential change in the year following new enrollment (mean marginal effect, −$387 [95% CI, −$1274 to $501) between groups. There were significant differences between groups in the change in spending on long-term services and supports, with maintained spending on community-based personal care services following new enrollment in D-SNPs compared with reductions for other MA, resulting in a relative increase of $343 (95% CI, $147 to $539).

**Conclusions and Relevance:**

This cohort study found that coordination-only D-SNPs was associated with maintained North Carolina Medicaid FFS spending levels for long-term services and supports compared with other MA plans, despite limited integration requirements. However, to reduce or delay nursing home transitions, higher levels of integration may be necessary.

## Key Points

**Question:**

For people who have dual eligibility for Medicare and Medicaid, does new enrollment in Dual-Eligible Special Needs Plans (D-SNPs) vs other Medicare Advantage (MA) change North Carolina Medicaid fee-for-service spending?

**Findings:**

This cohort study including 8869 individuals with D-SNP matched with 4389 individuals with other MA plans found that Medicaid spending on personal care services did not change following new enrollment into D-SNP compared with reductions following new enrollment in other MA plans; however, there was no significant difference in the change for total Medicaid spending between groups.

**Meaning:**

These findings suggest that dual-eligible Medicaid-Medicare beneficiaries enrolled in D-SNPs rather than other MA plans may benefit from increased use of long-term services and supports in their preferred care setting.

## Teaser

This cohort study examines changes in Medicaid fee-for-service spending before and after enrollment in coordination-only dual-eligible Special Needs Plans vs in other Medicare Advantage plans among community-living beneficiaries enrolled in both Medicare and Medicaid in North Carolina.

## Introduction

Medicare beneficiaries who are also enrolled in Medicaid (dual-eligible) drive significant spending in both Medicare and Medicaid programs.^1^ Their complex care needs and social risk factors are associated with high care utilization.^2^ Lack of care coordination and misaligned incentives across Medicare- and Medicaid-covered services drive inefficiencies that contribute to high costs among beneficiaries enrolled in both programs.^3^ Dual-eligible beneficiaries and their care partners often struggle to navigate 2 programs.^4^ To improve coordination, 46 state Medicaid programs contract with Dual Special Needs Plans (D-SNPs), a type of Medicare Advantage (MA) plan tailored for dual-eligible beneficiaries.^2,4,5^

D-SNPs provide the infrastructure for care coordination of services across both programs, and aim to prevent unnecessary use of high-cost services.^6,7,8,9^ Unlike other MA plans, all D-SNPs are required to contract with state Medicaid agencies, outline key coordination and align responsibilities across Medicare and Medicaid, and adhere to a model of care approved by the Centers for Medicare & Medicaid Services and the state Medicaid agency.^6^ As a result of this engagement with Medicaid programs, D-SNPs require states to have greater administrative capacity for contracting and oversight compared with other, non–D-SNP, MA plans.^10^ In addition to providing robust care coordination, some D-SNP contracts are also highly or fully financially integrated, meaning they are responsible for the cost of Medicaid services as well as Medicare services for beneficiaries enrolled in aligned plans.^10^

Consistent with evidence evaluating MA plans generally, D-SNPs have been found to be associated with reduced Medicare spending compared with traditional Medicare; however, differences between D-SNPs and other MA plans have been mixed.^11,12,13,14,15,16,17^ While D-SNPs seek efficiencies through care coordination, reductions in spending may indicate reduced access or quality of care. Medicaid programs and the Centers for Medicare & Medicaid Services often seek to shift spending to preventive services and lower-acuity care settings, such as a home or community setting.^18^ Understanding the impact of different types of D-SNP contracts on Medicaid spending is key for state administrators to improve care for dual-eligible beneficiaries.

Coordination-only D-SNP contracts continue to be the primary strategy for most states, and most D-SNP enrollees are in coordination-only contracts.^19^ Of the 46 states contracting with D-SNPs in 2023, 37 of them use coordination-only contracts, and 18 have highly or fully integrated contracts.^20,21^ Full integration requires comprehensive Medicaid-managed care, which is only available for dual-enrolled beneficiaries in 28 states as of 2023; thus, coordination D-SNPs are likely to continue to play a role in care for a substantial portion of the country. Despite states’ investment in coordination-only D-SNP contracting and oversight, it is plausible that coordination-only D-SNPs have no benefit or impact on Medicaid service use due to limited financial accountability; on the other hand, model of care requirements are intended to support appropriate service use, which includes home and community-based services provided by Medicaid.

States need evidence about whether or not coordination-only D-SNP contracts have the intended effect. Our objective was to understand whether coordination-only D-SNPs change use of Medicaid long-term services and supports compared with other types of MA plans. We do this by comparing the changes in North Carolina Medicaid Fee-for-Service (FFS) spending for people newly enrolled in coordination-only D-SNPs vs other MA plans among community-based full-benefit dual-eligible (FBDE) beneficiaries. North Carolina is an ideal case study because 95% of North Carolina Medicaid beneficiaries receive services through the FFS Medicaid program. Of dual-eligible beneficiaries, 1 in 3 were enrolled in coordination-only D-SNPs during the study period, the only type of D-SNP available in North Carolina.^1,22^ We expected D-SNPs to increase preventive care and reduce facility-based services through improved data sharing and care coordination. We hypothesized that Medicaid FFS spending would be lower for facility-based care but higher for community-based care after new enrollment in coordination-only D-SNPs compared with other MA plans among community-based FBDE beneficiaries.

## Methods

This cohort study was approved by the Duke University School of Medicine institutional review board and the North Carolina Department of Health and Human Services. The institutional review board determined this research to be exempt (45 CFR 46 104 [d][4]) from informed consent because it was considered to pose minimal risk. This study adhered to the Strengthening the Reporting of Observational Studies in Epidemiology (STROBE) reporting guideline for cohort studies (eFigure 1 in Supplement 1).

We compared changes in outcomes before and after enrollment for FBDE beneficiaries newly enrolled in coordination-only D-SNPs with those newly enrolled in other MA plans. The study was limited to North Carolina, which does not have any highly or fully financially integrated SNPs. Selection bias was a concern because beneficiaries may choose to enroll or not enroll in D-SNPs, and these choices may may be associated with patient outcomes.^23,24,25^ To address potential bias, we identified a control group with similar selection factors: FBDE beneficiaries newly enrolled in other MA plans. Assuming common unobserved factors driving selection into MA, the new-user, active comparator design can control for measured and unmeasured confounders.^26,27^

Our primary assumption was that the unobserved beneficiary factors driving selection into D-SNPs are similar to other MA plans.^28,29^ Observed factors were more similar between MA and D-SNP enrollees than compared with continuous FFS enrollees (eTable in Supplement 1). Both treated and control groups were dual-eligible for Medicare and Medicaid, so factors like socioeconomic status and access to health care practitioners were also comparable across the groups. D-SNPs and other MA are often owned by the same insurers, which compete in a similar market environments with similar clinician networks and limited differentiation in cost-sharing (due to Medicaid paying for all Medicare cost-sharing). Thus, D-SNPs and other MA plans are often perceived as similar by beneficiaries. Additionally, the assumption of parallel trends was tested using a 2-way fixed effects model estimating quarterly outcomes in the year prior to MA enrollment, and we did not find evidence of differential trends in our primary outcome, Medicaid FFS spending (eFigure 2 in Supplement 1). We mitigated residual observed confounding through propensity score weights as well as fixed effects for treatment group, region, and year.

### Sample

Our cohort consisted of community-living FBDE beneficiaries in North Carolina enrolled in FFS Medicare for 365 days before enrolling in either a D-SNP or another MA plan between 2014 and 2017. Medicare beneficiaries with partial Medicaid benefits, such as Medicare cost-sharing only, were excluded from our cohort, since Medicaid does not provide reimbursement for their care through the FFS payments. We excluded individuals enrolled in Program of All-inclusive Care for the Elderly (1144 individuals), those with fewer than 12 months of continuous Medicare FFS enrollment (119 291 individuals), individuals without FBDE status in the postenrollment period (7466 individuals), and those residing in nursing homes (8600 individuals) or receiving hospice care (2528 individuals). Of 194 211 individuals remaining, 13 258 had new enrollment in D-SNP or other MA during the period. Thus, the final analytic cohort included less than 4% of all FBDE beneficiaries in the state.

### Outcomes

A linkage^30^ between 100% Medicare and North Carolina Medicaid FFS claims data spanning from 2014 to 2017^1^ allowed us to identify health care services, eligibility, and patient characteristics across both payers before MA enrollment. After MA enrollment, we observed payments for Medicaid-funded services and supplemental Medicaid payments for Medicare-funded services.

The primary outcome of our study was Medicaid FFS spending, annualized to estimate rates per person-year (PPY) in the year before and after enrollment in D-SNPs or other MA plans. Total Medicaid expenditure was adjusted to 2017 US dollars using the Consumer Price Index medical component. We calculated total spending per person as well as spending by care settings aligned with Medicare claim types,^31^ including inpatient, outpatient facility, carrier, home health, hospice, durable medical equipment, postacute care, and long-term care. In our Medicaid data, we were unable to differentiate between postacute and long-term care skilled nursing facility payments; thus, our outcomes measure included both types of Medicaid spending on skilled nursing facility care. We created 2 additional categories of types of care in which Medicaid was the primary payer: personal care services (Current Procedural Terminology code 99509) and behavioral health services, as in prior work.^1^ Medicaid spending amounts were summed and divided by the time observed for each beneficiary to calculate the cost rate. To reflect intent-to-treat causal effect estimates, time following new enrollment was censored at the earliest date 1 year after new MA enrollment, loss of dual enrollment status, or end of study period (December 31, 2017).

### Covariates

We required 1 year of continuous FFS enrollment to measure baseline characteristics with Medicare FFS claims data. Self-reported race and ethnicity (Hispanic of any race, non-Hispanic Black, non-Hispanic White, non-Hispanic other race [eg, American Indian or Alaska Native, Asian, American Indian or Alaska Native, Native Hawaiian or Pacific Islander, and multiracial], unreported), age, age squared, sex, county rurality (defined as >70% rural residents), North Carolina Medicaid Home and community-based waiver program enrollment, use of personal care services, number of chronic conditions, Area Deprivation Index (ie, within-state decile ranking specified as continuous; range, 1-10, with higher score indicating greater disadvantage), Medicaid eligibility program (Qualified Medicare Beneficiary-Plus aged, Qualified Medicare Beneficiary-Plus blind or disabled, Specified Low-Income Medicare Beneficiary-Plus aged, Specified Low-Income Medicare Beneficiary-Plus blind or disabled, other Medicare age-eligible, other blind or disabled eligible, other Medicaid program), original eligibility for Medicare (disability or age), and year of new D-SNP or other MA enrollment were included in the final model. Race and ethnicity were included in the analysis to address selection bias because they are associated with D-SNP enrollment as well as outcomes. Medicaid data were considered the criterion standard, and Medicare data were used to impute missing values, resulting in very low missingness.^31^

### Statistical Analysis

We used inverse probability of treatment weighting to improve the comparability between groups. The calculated inverse probability of treatment score was used in weighting to generate the average treatment effect on the treated (ATT) for all analyses.^30,32^ Balance was assessed using standardized mean differences by treatment group at a less than 0.1 threshold.

Cost rates were presented as the point estimate with 95% CIs and weighted using the ATT estimates. For outcomes where less than 20% of beneficiaries had $0 costs (including total cost of care aggregating all care types across Medicaid and cost of care for claims submitted by physicians and other health care professionals), we used a generalized estimating equation with a γ distribution and beneficiary-level clustering. For outcomes characterized by high rates (≥20%) of zero spending, we used marginalized 2-part models with a log-skew-normal distribution as the best fit.^33,34^ For all models, we estimated rate ratios for the difference-in-differences spending and calculated the mean marginal effect using the method of recycled estimations to isolate the impact of being a new D-SNP enrollment vs new other MA enrollee on spending. *P* values were 2-sided, and statistical significance was set at *P* ≤ .05. Analyses were performed using STATA software version 17.0 (StataCorp). Data were analyzed from August 2023 to November 2024.

## Results

Among 8869 participants in the D-SNP cohort, 4762 (53.7%) were younger than age 65 years, 5833 (65.8%) were female, and 975 (11.0%) resided in rural areas. The sample included 333 Hispanic participants (3.8%), 3816 non-Hispanic Black participants (43.0%), 3978 non-Hispanic White participants (44.9%), 283 non-Hispanic other race participants (3.2%), and 459 participants with unreported race and ethnicity (5.2%). The D-SNP cohort was characterized by high comorbidity (median [IQR], 7.0 [4.0-9.0] chronic conditions) and used home- and community-based services waiver services (333 participants [3.8%]) and personal care services (1328 participants [15.0%]). Medicare original eligibility due to disability or blindness was prevalent (6155 participants [69.5%]). Area deprivation was relatively high compared with the rest of the state (70th percentile). Before ATT weighting, D-SNP enrollees were more likely to be non-Hispanic Black and live in nonrural areas than the sample of 4389 other MA enrollees (4706 [53.2%] aged <65 years; 5739 [64.9%] female; 971 [11.0%] rural); however, once weighted, all baseline absolute standardized differences were less than 0.1, indicating good balance (Table 1).

**Table 1.** Differences in Baseline Characteristics Between Medicare-Medicaid Dual-Enrolled Beneficiaries With New Enrollment in D-SNPs Compared With Other MA Plans (2014-2017)

New enrollee characteristics	No. (%)			Standardized difference after weighting, %
	D-SNP (n = 8869)	Other MA plans		
		Unweighted (n = 4389)	Weighted (n = 4389)^a^	
Age, median (IQR), y	63.0 (54.0-70.0)	61.0 (47.0-71.0)	63.0 (53.0-70.0)	0.4
Chronic conditions, median (IQR)	7.0 (4.0-9.0)	6.0 (3.0-9.0)	7.0 (4.0-9.0)	0.7
ADI state decile, median (IQR)	7.0 (5.0-9.0)	7.0 (4.0-9.0)	7.0 (5.0-8.0)	1.3
Age group				
Pediatric and adult (<65 y)	4762 (53.7)	2448 (55.8)	4706 (53.2)	1.0
Older adult (≥65 y)	4107 (46.3)	1941 (44.2)	4138 (46.8)	
Race and ethnicity^b^				
Hispanic	333 (3.8)	243 (5.5)	321 (3.6)	1.3
Non-Hispanic Black	3816 (43.0)	1534 (35.0)	3812 (43.1)	
Non-Hispanic White	3978 (44.9)	2250 (51.3)	3968 (44.9)	
Non-Hispanic other race	283 (3.2)	155 (3.5)	269 (3.0)	
Unreported	459 (5.2)	207 (4.7)	474 (5.4)	
Sex				
Male	3036 (34.2)	1702 (38.8)	3105 (35.1)	1.8
Female	5833 (65.8)	2687 (61.2)	5739 (64.9)	
Rural county of residence				
Urban	7894 (89.0)	3166 (72.1)	7873 (89.0)	0.1
Rural	975 (11.0)	1223 (27.9)	971 (11.0)	
1915(c) Waivers (CAP_DA/C and Innovations/TBI)	333 (3.8)	135 (3.1)	332 (3.8)	0.0
Personal care Services use	1328 (15.0)	456 (10.4)	1322 (14.9)	0.1
Medicaid current eligibility category: QMB+ blind or disabled	4421 (49.8)	2097 (47.8)	4306 (48.7)	2.3
Disabled or blind as original reason for Medicare eligibility	6166 (69.5)	3022 (68.9)	6133 (69.3)	0.4
Index year				
2015	2947 (33.2)	1527 (34.8)	3113 (35.2)	4.2
2016	2804 (31.6)	1251 (28.5)	2714 (30.7)	
2017	3118 (35.2)	1611 (36.7)	3018 (34.1)	

Abbreviations: ADI, Area Deprivation Index; D-SNP, dual-eligible Special Needs Plans; MA, Medicare Advantage; TBI, Traumatic Brain Injury.

^a^ Characteristics for the new enrollees in other MA plans are adjusted using inverse propensity score weights for average treatment effect on the treated (ATT).

^b^ Hispanic ethnicity includes all racial groups. Non-Hispanic other race includes American Indian or Alaska Native, Asian, Native Hawaiian or Pacific Islander, and multiracial. Unreported includes those with unknown race or ethnicity.

### ATT-Weighted Medicaid Spending Rates by Treatment Group

In the year before new enrollment (Table 2), mean Medicaid FFS spending for the D-SNP cohort was $7145 (95% CI, $6760-$7530) PPY. Key cost drivers included outpatient facility services at $4031 (95% CI, $3691-$4372), carrier services at $2826 (95% CI, $2673-$2979), and personal care services at $1446 (95% CI, $1367-$1525). Spending at baseline was similar for the other MA cohort after weighting, except higher nursing facility care among the other MA cohort ($763 [95% CI, $596-$930]) compared with the D-SNP cohort ($127 [95% CI, $85-$169]).

**Table 2.** North Carolina Medicaid Fee-for-Service Spending Rates PPY in the Year Before New Enrollment in D-SNP or Other MA Plan (2014-2017)

Category	Spending PPY in the year before new enrollment^a^	
	New enrollees in D-SNP, mean (95% CI), $	New enrollees in Other MA Plan, mean (95% CI), $ (weighted)
Total	7145 (6760-7530)	7732 (7125-8339)
By care settings aligned with Medicare claim types		
Inpatient	37 (25-49)	59 (41-78)
Outpatient facility	4031 (3691-4372)	4021 (3498-4543)
Carrier	2826 (2673-2979)	2670 (2471-2870)
Home health	106 (79-132)	169 (106-231)
Nursing facility care^b^	127 (85-169)	763 (596-930)
DME	19 (8-30)	40 (2-77)
Subset of key services for which Medicaid is primary payer		
Personal care services	1446 (1367-1525)	1414 (1303-1525)
Behavioral health services	799 (708-890)	677 (563-792)

Abbreviations: D-SNP, dual-eligible Special Needs Plans; DME, Durable Medical Equipment; MA, Medicare Advantage; PPY, per person year.

^a^ Rates shown as cost PPY in 2017 US dollars. Medicaid spending rates for the new enrollees in other MA plans are adjusted using inverse propensity score weights for average treatment effect on the treated.

^b^ Nursing facility care includes Medicaid spending for long-term care in nursing facilities as well as supplemental payments for postacute care in nursing facilities for stays in which Medicare was the primary payer.

**Figure 1.**
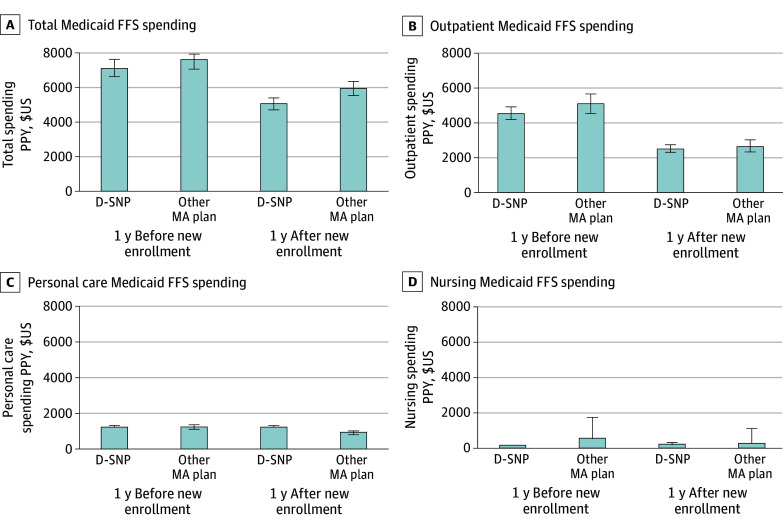
North Carolina Medicaid Fee-for-Service (FFS) Adjusted Spending Rates per Person-Year (PPY) in the Year Before and After New Enrollment in Dual-Eligible Special Needs Plans (D-SNP) or Other Medicare Advantage (MA) Plans Source: linked 100% Medicare and North Carolina Medicaid claims data 2014 to 2017 for Medicare FFS beneficiaries enrolled in North Carolina Medicaid. Results shown are estimations from adjusted regression models. All models use inverse propensity score weights for average treatment effect on the treated in the intent-to-treat primary analysis and are adjusted for index year and region. Total and carrier costs are estimated using generalized estimating equations with a γ distribution and account for beneficiary-level clustering. The remaining models are estimated using a 2-part model with log-skew-normal distribution due to having more than 20% of observations with $0 costs.

Similarly, estimated Medicaid FFS spending PPY from the weighted regression models adjusted for region and year (Figure 1) was not significantly different between treatment groups prior to new enrollment, except for nursing facility spending. In the year following new enrollment, mean Medicaid FFS spending reduced for both D-SNP ($5102 [95% CI, $4801-$5340] PPY) and other MA ($5986 [95% CI, $4925-$7047] PPY) cohorts. Estimated spending for key cost drivers, for example outpatient care, was also reduced for both D-SNP and MA groups in the year following new enrollment compared with the year prior. As expected, enrollment in managed care across both treatment groups was associated with reduced spending in many cost categories; however, enrollees experienced smaller declines in Medicaid personal care services spending following enrollment in D-SNPs than in other MA plans.

### Mean Marginal Effects From Adjusted Models and ATT Weighting

**Figure 2.**
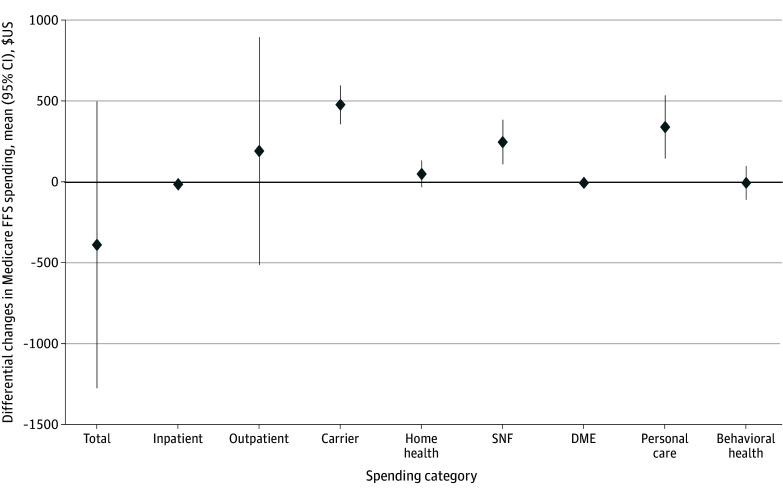
Mean Marginal Effect on Estimated North Carolina Medicaid Fee-for-Service (FFS) Spending for New Dual-Eligible Special Needs Plans Enrollment Compared With New Other Medicare Advantage Enrollment Among Medicare-Medicaid Dual-Enrolled Beneficiaries Source: linked 100% Medicare and North Carolina Medicaid claims data 2014 to 2017 for Medicare FFS beneficiaries enrolled in North Carolina Medicaid. The mean marginal effect is estimated as the difference in the change in expected Medicaid spending in the year following new enrollment compared with the prior year for dual-eligible Special Needs Plans enrollees compared with other Medicare Advantage enrollees. All models use inverse propensity score weights for average treatment effect on the treated in the intent-to-treat primary analysis and are adjusted for index year and region. Total and carrier costs are estimated using generalized estimating equations with a γ distribution and account for beneficiary-level clustering. The remaining models are estimated using a 2-part model with log-skew-normal distribution due to having more than 20% of observations with $0 costs. The outpatient model is adjusted for region only and the behavioral health model is unadjusted to achieve model convergence. DME indicates durable medical equipment; SNF, skilled nursing facility.

There was no significant difference in the change for total Medicaid FFS spending PPY (mean marginal effect, −$387 [95% CI, −$1275 to $501]) among the D-SNP cohort compared with other MA (Figure 2). Compared with the other MA plans cohort, the D-SNP cohort had significantly different increases in carrier spending by $480 (95% CI, $360 to $600) or 23% (adjusted rate ratio [aRR], 1.23 [95% CI, 1.17 to 1.28), nursing facility care spending by $250 (95% CI, $112 to $387) PPY or 86% (aRR, 1.86 [95% CI, 1.21 to 2.85]), and personal care services spending by $343 (95% CI $147 to $539]) or 38% (aRR, 1.38 [95% CI, 1.15 to 1.65]) (eFigure 3 and eFigure 4 in Supplement 1). However, the low baseline spending levels on nursing facility care in the D-SNP cohort limited the potential for large declines. The differential reduction in inpatient spending among D-SNP enrollees compared with other MA enrollees was not significant, and there was a low prevalence of any Medicaid payments for inpatient care across both cohorts, particularly in the postenrollment period (2.7%). Results in sensitivity analyses (as treated censoring, winsorized costs) were consistent with the primary analysis (eFigure 5 and eFigure 6 in Supplement 1).

## Discussion

This cohort study of coordination-only D-SNPs in North Carolina found some evidence that changes in Medicaid spending were different following new enrollment coordination-only D-SNPs compared with other MA plans. As expected, enrollment in managed care across both treatment groups was associated with reduced spending in many cost categories; however, enrollees experienced smaller declines in personal care services spending following enrollment in D-SNPs than in other MA plans. This evidence suggests that requiring MA plans to contract with Medicaid programs to support care coordination for dual-eligible beneficiaries may impact the use of long-term services and supports in the first year after enrollment. However, if states seek to reduce or delay nursing home transitions, higher levels of integration may be needed.

These findings suggest that coordination-only D-SNPs may maintain access to Medicaid-funded long-term services and supports across care settings. In our study period, North Carolina D-SNPs were coordination-only contracts, which means they were not accountable for the cost of Medicaid services. Our findings for a relative increase in community-based personal care services is similar to findings in Minnesota and California, which have a mix of D-SNP contracts.^35,36,37^ Studies of Medicaid populations in other states have found more highly integrated D-SNP contracts are associated with reduced nursing home use; for example, studies in Tennessee and Massachusetts have found highly or fully financially integrated D-SNPs were associated with reduced nursing home use.^38,39^ While Tennessee has a mix of D-SNP contracts, including coordination-only, aligned enrollment in Tennessee grew from 7% to 24% over the period studied, which supported the evaluation of outcomes associated with aligned D-SNP enrollment.^38^ Similar to prior evidence, we found no evidence of an association between D-SNP and total Medicaid spending.^3,13,15,40^

Our study evaluates changes in Medicaid spending to inform decision-making for states seeking to leverage D-SNP contracts to shift their performance on home- and community-based services measures.^41,42^ In North Carolina, we observed low enrollment in D-SNPs among people living in nursing homes at baseline, leading to our decision to focus on the community-based population. Other integrated options are intentionally tailored to the needs of people with an institutional level of care, such as Program of All-inclusive Care for the Elderly or institutional SNPs; however, these are not widely scaled.^43,44^ This raises concerns about how to scale integrated payment models for people living in skilled nursing facilities.

While the promise of full integration is hopeful, currently most states have coordination-only contracts,^45^ and coordination-only contracts reduce the burden on states to support aligned enrollment, health care practitioner networks, and appeals and oversight processes.^10^ Coordination-only D-SNPs meet a need for coordinated care among FBDE beneficiaries who are served by an FFS state Medicaid program.^6^ By contrast, fully financially integrated SNP contracts require enrollment in capitated programs for both Medicare and Medicaid benefits.^46^ As a result, only 25% of dual-eligible beneficiaries nationally are eligible for fully financially integrated SNP enrollment.^47^ While greater financial integration is expected to incentivize insurers and clinicians to reduce unnecessary hospital and nursing home use, more evidence is needed to confirm its effectiveness and justify the additional investment in state oversight.^3,48,49,50^

### Limitations

This study has some limitations. Our findings were limited to the subgroup of beneficiaries newly enrolled in coordination-only D-SNPs or other MA plans (4% for this North Carolina study). Differences in the characteristics of this subgroup compared with the group with continuous Medicare FFS enrollment suggest these results may not generalize to the broader FBDE population. Medicare spending and use data were unavailable for this analysis due to a lack of Medicare encounter information for the period when beneficiaries are enrolled in MA. Thus, we were unable to evaluate whether changes in Medicaid spending reflect changes in total costs or utilization for services where Medicare is the primary payer. These findings may not generalize to dual enrollees in states beyond North Carolina. The prevalence of eligibility groups, FFS Medicaid enrollment, and participation in waivers may vary across states due to differences in state programs and coverage policies. For instance, North Carolina offers access to personal care services through 1115, 1915(b), and 1915(c) waivers for integrated mental health, home- and community-based services), and individuals with intellectual and developmental disabilities.^51^ Moreover, while our study design controlled for differences between groups that remain constant during the study period, there remains potential for bias due to unobserved, time-varying confounders that may differentially impact the treated and control group.

## Conclusions

In this cohort study of coordination-only D-SNPs in North Carolina, we found that D-SNPs were associated with differences in how Medicaid dollars were spent compared with other MA plans; however, if states seek to reduce or delay nursing home transitions, higher levels of integration may be needed. State Medicare-Medicaid integration efforts may further incentivize D-SNPs to improve care coordination and outcomes for beneficiaries.

## Notes

Supplement 1. **eFigure 1.** Diagram of Cohort Selection Using Linked Medicare-Medicaid Data 2014-2017 **eTable.** Baseline Characteristics of Medicare Beneficiaries Dual-Enrolled in North Carolina Medicaid (Unweighted) 2014-2017 **eFigure 2.** Comparison of Trends in Total Medicaid Fee-for-Service Spending in the Year Prior to D-SNP or Other MA Enrollment, Weighted Intent-to-Treat **eFigure 3.** Ratio Comparing Odds of Any Medicaid Spending in the Year Following New Enrollment in D-SNP and Other MA Compared to the Prior Year, Intent-to-Treat Primary Analysis **eFigure 4.** Ratios Comparing Changes in Medicaid Spending Rates in the Year Following New Enrollment in D-SNP vs Other MA Compared to the Prior Year, Intent-to-Treat Primary Analysis **eFigure 5.** Sensitivity Analysis: As-Treated AME for Enrollment in D-SNP vs Other MA Plan, Non-Winsorized **eFigure 6.** Sensitivity Analysis: Intent-to-Treat Average Marginal Effects for Enrollment in D-SNP vs Other MA Plan, Winsorized

Supplement 2.
Data Sharing Statement
