# Supplementary material for: Medicaid Spending in Coordination-Only Dual-Eligible Special Needs Plans
Source: JAMA Netw Open. 2025 Jan 22;8(1):e2455461. doi: 10.1001/jamanetworkopen.2024.55461 (PMC11755192; doi:10.1001/jamanetworkopen.2024.55461)
Supplement: Supplement 1. — eFigure 1. Diagram of Cohort Selection Using Linked Medicare-Medicaid Data 2014-2017 eTable. Baseline Characteristics of Medicare Beneficiaries Dual-Enrolled in North Carolina Medicaid (Unweighted) 2014-2017 eFigure 2. Comparison of Trends in Total Medicaid Fee-for-Service Spending in the Year Prior to D-SNP or Other MA Enrollment, Weighted Intent-to-Treat eFigure 3. Ratio Comparing Odds of Any Medicaid Spending in the Year Following New Enrollment in D-SNP and Other MA Compared to the Prior Year, Intent-to-Treat Primary Analysis eFigure 4. Ratios Comparing Changes in Medicaid Spending Rates in the Year Following New Enrollment in D-SNP vs Other MA Compared to the Prior Year, Intent-to-Treat Primary Analysis eFigure 5. Sensitivity Analysis: As-Treated AME for Enrollment in D-SNP vs Other MA Plan, Non-Winsorized eFigure 6. Sensitivity Analysis: Intent-to-Treat Average Marginal Effects for Enrollment in D-SNP vs Other MA Plan, Winsorized [file jamanetwopen-e2455461-s001.pdf]

Supplemental Online Content

Coulibaly N, Jones KA, Smith VA, et al. Medicaid spending in coordination-only dual-eligible Special Needs Plans. JAMA Netw Open. 2025;8(1):e2455461. doi:10.1001/jamanetworkopen.2024.55461

eFigure 1. Diagram of Cohort Selection Using Linked Medicare-Medicaid Data 2014-2017

eTable. Baseline Characteristics of Medicare Beneficiaries Dual-Enrolled in North Carolina Medicaid (Unweighted) 2014-2017

eFigure 2. Comparison of Trends in Total Medicaid Fee-for-Service Spending in the Year Prior to D-SNP or Other MA Enrollment, Weighted Intent-to-Treat

eFigure 3. Ratio Comparing Odds of Any Medicaid Spending in the Year Following New Enrollment in D-SNP and Other MA Compared to the Prior Year, Intent-to-Treat Primary Analysis

eFigure 4. Ratios Comparing Changes in Medicaid Spending Rates in the Year Following New Enrollment in D-SNP vs Other MA Compared to the Prior Year, Intent-to-Treat Primary Analysis

eFigure 5. Sensitivity Analysis: As-Treated AME for Enrollment in D-SNP vs Other MA Plan, Non-Winsorized

eFigure 6. Sensitivity Analysis: Intent-to-Treat Average Marginal Effects for Enrollment in D-SNP vs Other MA Plan, Winsorized

This supplemental material has been provided by the authors to give readers additional information about their work.

© 2024 Coulibaly N et al. *JAMA Network Open*.

eFigure 1. Diagram of cohort selection using linked Medicare-Medicaid data 2014-2017

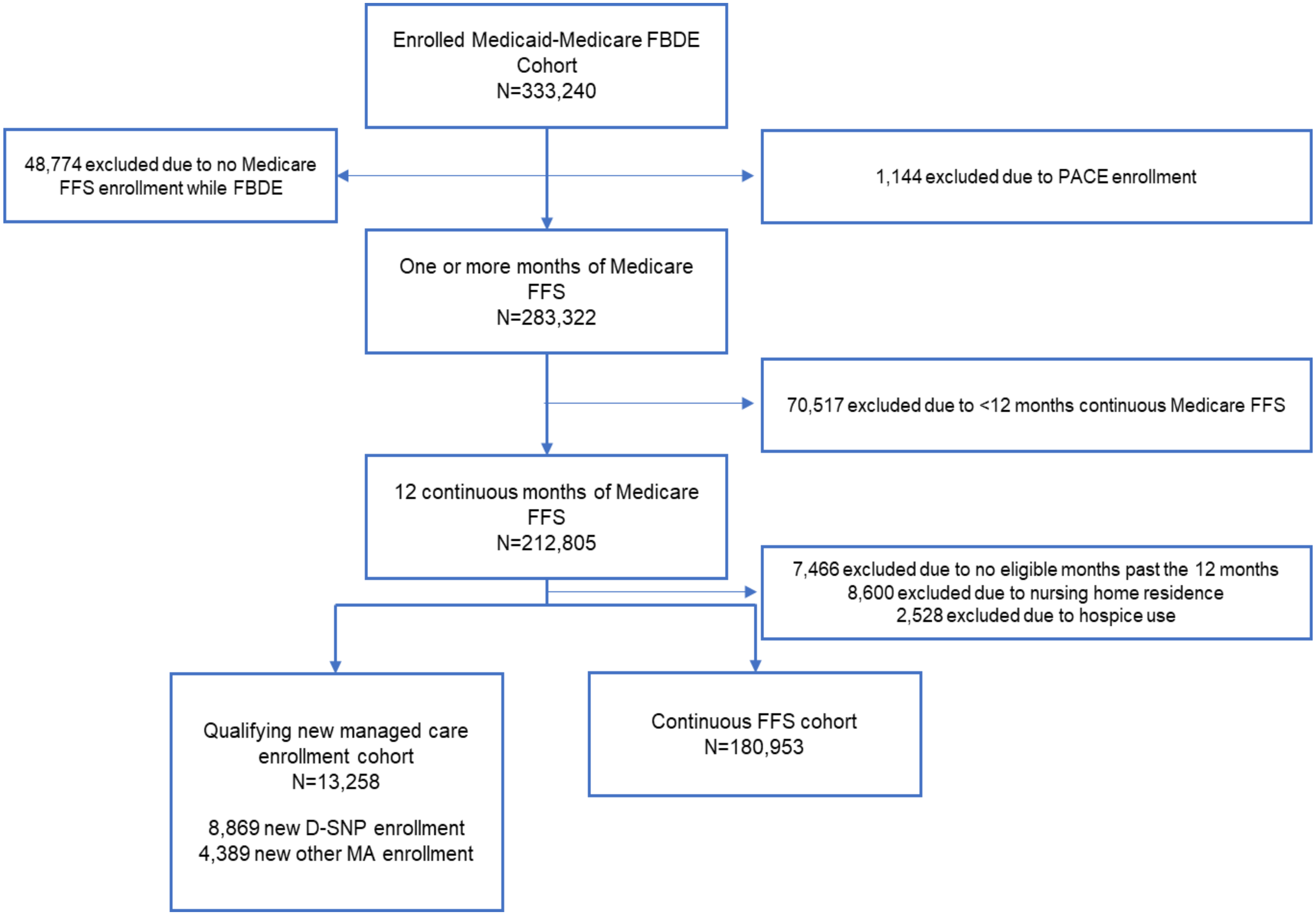

D-SNP, dual eligible special needs plan; FBDE=full benefit dual eligible; FFS=fee-for-service; MA, Medicare Advantage; PACE, Program of All-Inclusive Care for the Elderly

eTable 1. Baseline characteristics of Medicare beneficiaries dual-enrolled in North Carolina Medicaid (Unweighted) 2014-2017

© 2024 Coulibaly N et al. *JAMA Network Open*.

	Post Period Medicare Plan Category			
	D-SNP new enrollee (*N* = 8,869)	Other MA new enrollee Unweighted (*N* = 4,389)	Continuous Medicare FFS (*N = 180,953)*	All
Age (years), Median (Q1, Q3)	63.0 (54.0, 70.0)	61.0 (47.0, 71.0)	64.0 (50.0, 74.0)	64.0 (50.0, 74.0)
Age group				
Pediatric and Adult (<65 years)	4,762 (53.7%)	2,448 (55.8%)	90,661 (50.1%)	97,871 (50.4%)
Aged Adult (65 years and over)	4,107 (46.3%)	1,941 (44.2%)	90,292 (49.9%)	96,340 (49.6%)
Race and Ethnicity				
Non-Hispanic White	3,978 (44.9%)	2,250 (51.3%)	96,835 (53.5%)	103,063 (53.1%)
Non-Hispanic Black	3,816 (43.0%)	1,534 (35.0%)	59,771 (33.0%)	65,121 (33.5%)
Hispanic	333 (3.8%)	243 (5.5%)	6,303 (3.5%)	6,879 (3.5%)
Non-Hispanic other race	283 (3.2%)	155 (3.5%)	8,107 (4.5%)	8,545 (4.4%)
Unreported	459 (5.2%)	207 (4.7%)	9,937 (5.5%)	10,603 (5.5%)
Female sex	5,833 (65.8%)	2,687 (61.2%)	109,215 (60.4%)	117,735 (60.6%)
Rural county of residence	975 (11.0%)	1,223 (27.9%)	66,510 (36.8%)	68,708 (35.4%)
Chronic conditions, Median (Q1, Q3)	7.0 (4.0, 9.0)	6.0 (3.0, 9.0)	6.0 (3.0, 9.0)	6.0 (3.0, 9.0)
1915(c) Waivers (CAP_DA/C and Innovations/TBI)	333 (3.8%)	135 (3.1%)	9,962 (5.5%)	10,430 (5.4%)
Personal Care Services use	1,328 (15.0%)	456 (10.4%)	25,734 (14.2%)	27,518 (14.2%)
Medicaid current eligibility category: QMB+ blind/disabled	4,421 (49.8%)	2,097 (47.8%)	76,690 (42.4%)	83,208 (42.8%)
Disabled/blind as original reason for Medicare eligibility	6,166 (69.5%)	3,022 (68.9%)	113,984 (63.0%)	123,172 (63.4%)
Index year				
2015	2,947 (33.2%)	1,527 (34.8%)	143,143 (79.1%)	147,617 (76.0%)
2016	2,804 (31.6%)	1,251 (28.5%)	20,551 (11.4%)	24,606 (12.7%)
2017	3,118 (35.2%)	1,611 (36.7%)	17,259 (9.5%)	21,988 (11.3%)
ADI state rank, Median (Q1, Q3)	7.0 (5.0, 9.0)	7.0 (4.0, 9.0)	7.0 (5.0, 9.0)	7.0 (5.0, 9.0)

[a]Hispanic ethnicity includes all racial groups. Non-Hispanic other race includes Asian, American Indian/Alaska Native, Native Hawaiian/Pacific Islander, and multiracial. Unreported includes those with unknown race and/or ethnicity.

Source: Authors' analysis of linked 100% Medicare and NC Medicaid claims data 2014-2017 for Medicare fee-for-service community-based beneficiaries enrolled in NC Medicaid. Cohort includes Medicaid beneficiaries with at least one year of continuous Medicare fee-for-service to evaluate baseline characteristics.

eFigure 2. Comparison of trends in Medicaid fee-for-service spending in the year prior to D-SNP or other MA enrollment, Weighted intent-to-treat (ITT) and p-value for test of parallel trends is shown.

© 2024 Coulibaly N et al. *JAMA Network Open*.

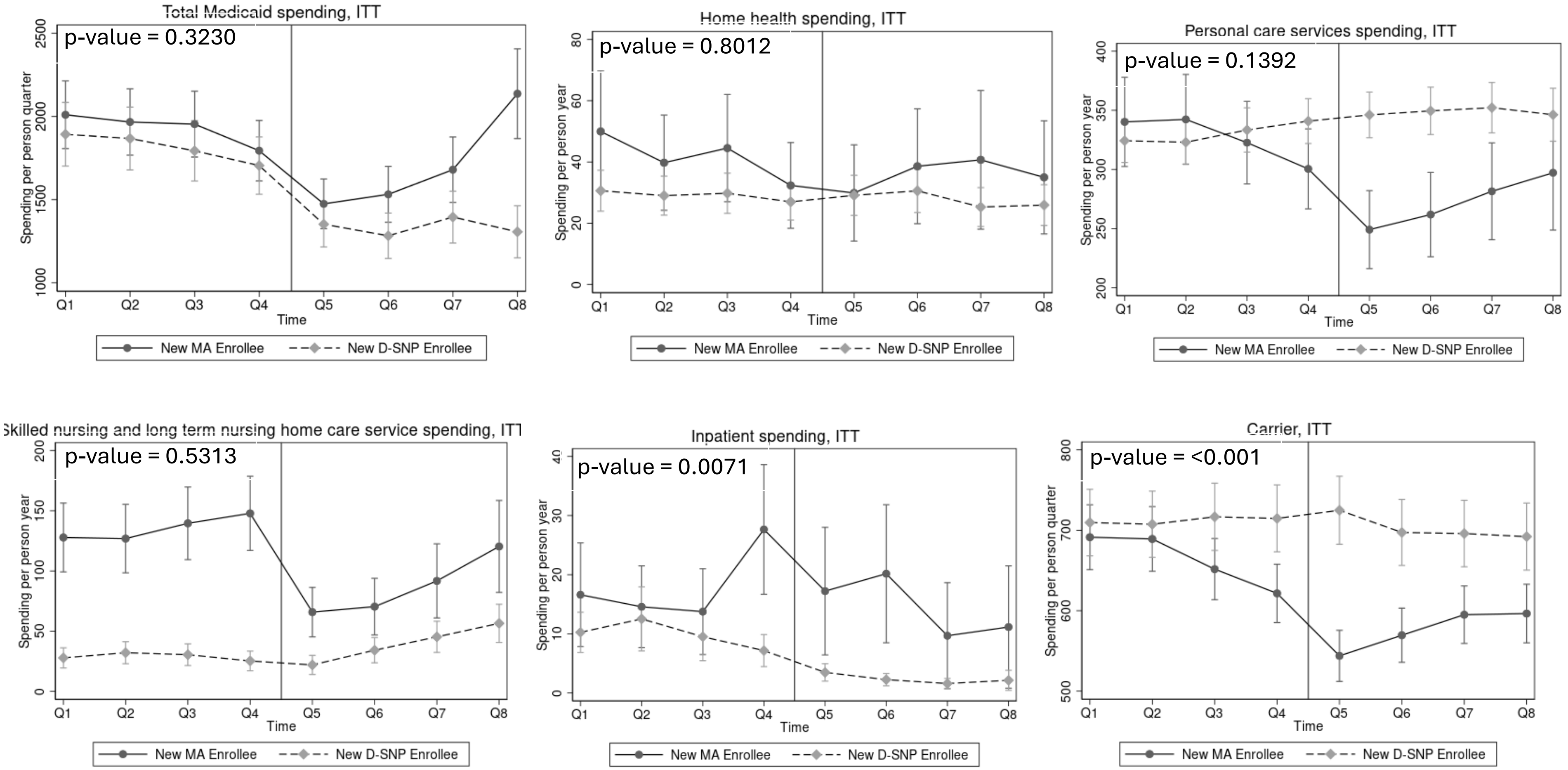

© 2024 Coulibaly N et al. *JAMA Network Open*.

eFigure 3. For outcomes using two-part models: Ratio comparing odds of any Medicaid spending in the year following new enrollment in D-SNP and Other MA compared to the prior year, intent-to-treat primary analysis (estimated using ATT weights in adjusted models)

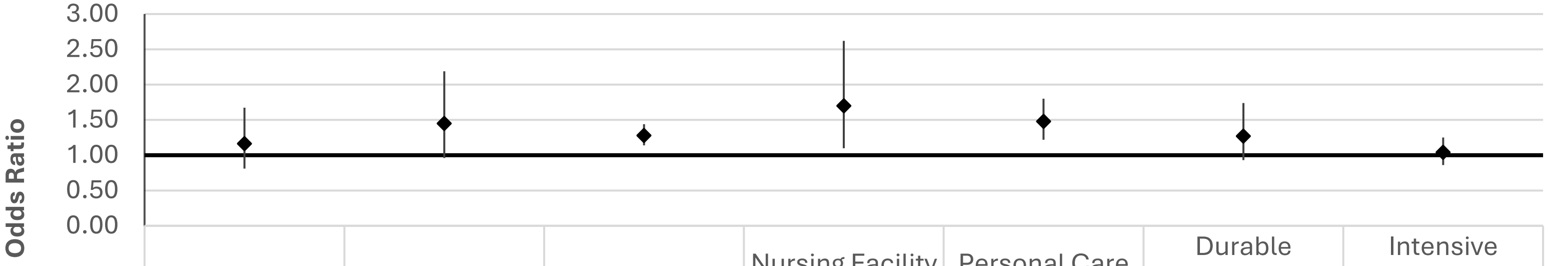

	Inpatient	Home Health	Outpatient	Nursing Facility Care	Personal Care Services (LTSS)	Durable Medical Equipment	Intensive Behavioral Health
Upper CI limit	1.67	2.19	1.44	2.62	1.80	1.74	1.25
Lower CI limit	0.81	0.96	1.14	1.10	1.22	0.93	0.86
◆ Estimate	1.16	1.45	1.28	1.70	1.48	1.27	1.04

eFigure 4. Ratios comparing changes in Medicaid spending rates in the year following new enrollment in D-SNP vs Other MA compared to the prior year, intent-to-treat primary analysis (estimated using ATT weights in adjusted models)

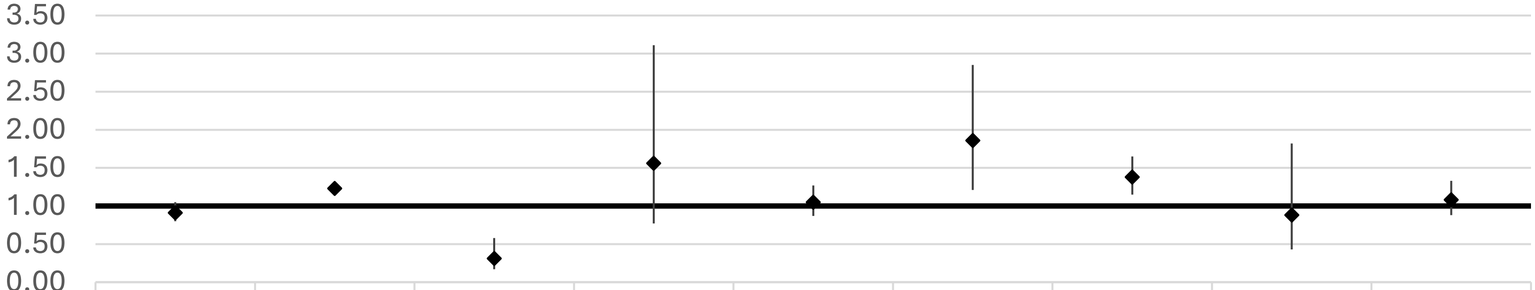

	Total	Carrier	Inpatient	Home Health	Outpatient	Nursing Facility Care	Personal Care	DME	Behavioral Health
Upper CI limit	1.05	1.28	0.58	3.11	1.27	2.85	1.65	1.82	1.33
Lower CI limit	0.80	1.17	0.17	0.77	0.87	1.21	1.15	0.43	0.88
◆ Estimate	0.91	1.23	0.31	1.56	1.05	1.86	1.38	0.88	1.08

CI=95% confidence interval; MA=Medicare Advantage; D-SNP= dual-eligible Special Needs Plans; DME=Durable Medical Equipment. Adjusted Rate Ratios estimated using two-part marginalized regression models.

© 2024 Coulibaly N et al. *JAMA Network Open*.

Sensitivity Analyses We performed two sets of sensitivity analyses. First, we winsorized the cost outcomes at the 99.95th percentile (based on cost distributions). Second, we used the as-treated time observed to calculate spending rates. Model estimation methods remained the same as in the primary analysis. All sensitivity model results were compared descriptively to the primary results.

eFigure 5. As-Treated AME for enrollment in D-SNP vs other MA Plan, non-winsorized

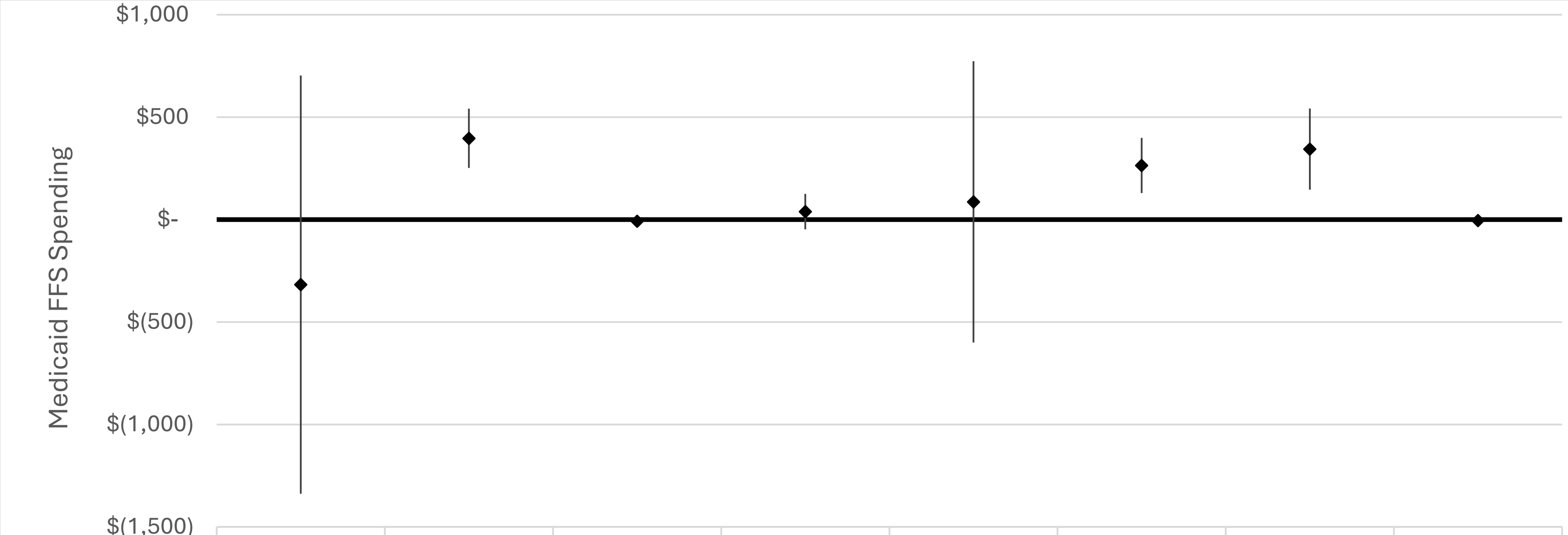

	Total Medicaid FFS Spending	Carrier	Inpatient	Home Health	Outpatient	Nursing Facility Care	Personal Care	Durable Medical Equipment
Upper CI limit	$704	$541	$22	$126	$773	$399	$542	$15
Lower CI limit	$(1,338)	$252	$(38)	$(48)	$(600)	$129	$146	$(24)
◆ Estimate	$(317)	$397	$(8)	$39	$86	$264	$344	$(5)

© 2024 Coulibaly N et al. *JAMA Network Open*.

eFigure 6. Intent-to-treat average marginal effects for enrollment in D-SNP vs other MA Plan, winsorized

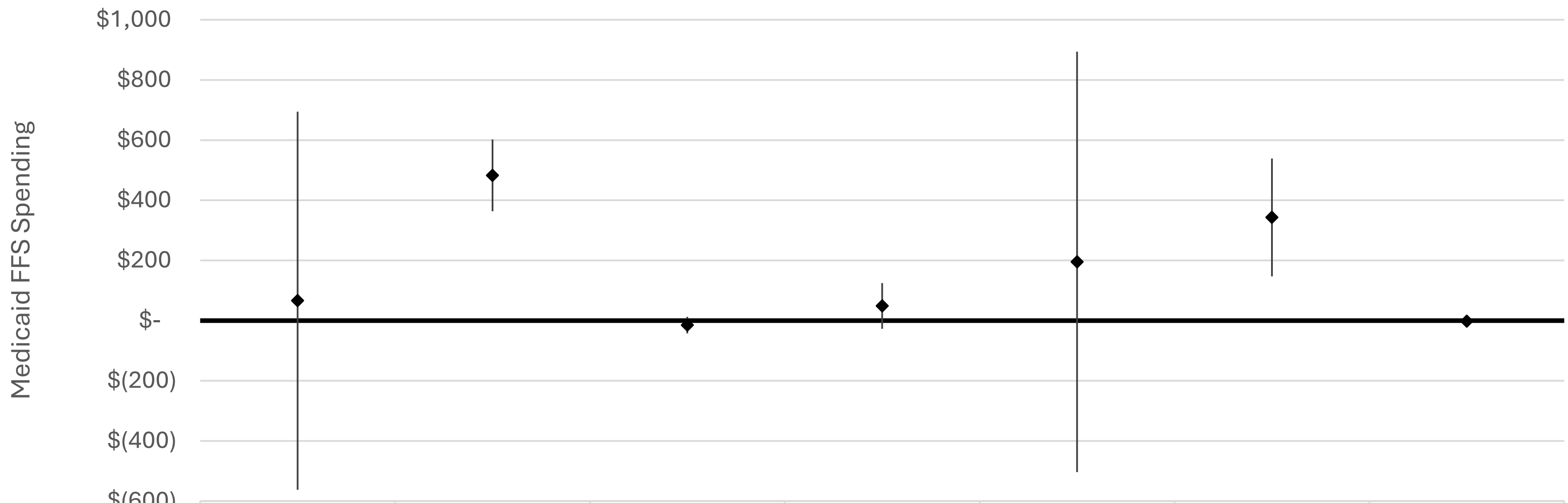

	Total Medicaid FFS Spending	Carrier	Inpatient	Home Health	Outpatient	Personal Care	Durable Medical Equipment
Upper CI limit	$695	$602	$13	$125	$894	$539	$11
Lower CI limit	$(562)	$364	$(43)	$(27)	$(504)	$147	$(15)
◆ Estimate	$66	$483	$(15)	$49	$195	$343	$(2)

Notes: Models predicting skilled nursing home and behavioral health spending did not converge.

© 2024 Coulibaly N et al. *JAMA Network Open*.
