# Supplementary material for: Medicaid Spending in Coordination-Only Dual-Eligible Special Needs Plans
Source: JAMA Netw Open. 2025 Jan 22;8(1):e2455461. doi: 10.1001/jamanetworkopen.2024.55461 (PMC11755192; doi:10.1001/jamanetworkopen.2024.55461)
Supplement: Supplement 2. — Data Sharing Statement [file jamanetwopen-e2455461-s002.pdf]

Data Sharing Statement

Coulibaly. Medicaid Spending in Coordination-Only Dual-Eligible Special Needs Plans. *JAMA Netw Open*. Published January 22, 2025. doi:10.1001/jamanetworkopen.2024.55461

Data
Data available: No

Additional Information
Explanation for why data not available: Data are unable to be shared due to restricted data use agreements.
